# Resilience Challenges in Bioeconomy Policies: A Global Comparative Analysis

**DOI:** 10.1007/s00267-026-02432-1

**Published:** 2026-04-09

**Authors:** Nicolai Goritz, Maria Proestou, Peter H. Feindt

**Affiliations:** 1 Head of the Research Group BIOPOLISTA, Humboldt-Universität zu Berlin, Thaer-Institut for Agricultural and Horticultural Sciences, Agricultural and Food Policy Group, Berlin, Germany; 2 Professor of Agricultural and Food Policy, Humboldt-Universität zu Berlin, Thaer-Institut for Agricultural and Horticultural Sciences, Agricultural and Food Policy Group, Berlin, Germany

**Keywords:** Bio-based economy, Issue salience, Bio-based production systems, Policy design, Sustainability, Systemic shocks

## Abstract

The sustainability of social-ecological systems has become a major concern in environmental policy and beyond, triggering many integrated cross-policy initiatives. An important example are bioeconomy strategies. Policies to promote a bio-based economy have been enacted in more than 50 countries around the world, often in response to the sustainability challenges posed by the fossil-based economy. However, the success and sustainability of the bioeconomy, in turn, depends on the resilience of the bio-based production systems on which it rests. Simultaneously, the continued delivery of the desired functions of these systems is potentially threatened by environmental, social, economic, and political short-term shocks and long-term stresses. Prudent bioeconomy policies would therefore identify potential disruptions and develop pro-active strategies to address them. However, little is known to what extent and which resilience challenges for a sustainable bioeconomy have been addressed in bioeconomy policies. To investigate the salience of resilience challenges in bioeconomy policies, we conducted a systematic content analysis of 78 national bioeconomy policy documents in 50 countries published between 2005 and 2020 to identify and discuss the specific challenges and the instruments directly aimed at addressing them. The results show that bioeconomy policy documents largely pay little attention to resilience challenges. If mentioned at all, challenges are mostly framed in economic terms, with few concrete instruments proposed to enhance resilience. Overall, the general neglect of resilience issues in global bioeconomy policymaking underscores the need for stronger foresight, cross-sector dialogue, and the explicit integration of resilience considerations into environmental management and bioeconomy policy design.

## Introduction

In the face of planetary boundaries (Rockström et al. 2023), a transformative shift from a fossil-based towards a bio-based economy has been proposed as a pathway to ensure a sustainable supply of food, fiber and renewable energy that would also help to address climate, biodiversity, economic, and health problems (Scarlat et al. 2015; Murray et al. 2017; IACGB 2020; Dolge et al. 2023; Eversberg et al. 2023; Albay et al. 2025). Policy statements which promote such a transformation have proliferated around the world, as exemplified by the widespread adoption of holistic (or dedicated) bioeconomy strategies and bioeconomy-related policies by some 50 countries (Meyer 2017; Dietz et al. 2018; Vogelpohl et al. 2021; Proestou et al. 2024).

Although there is no single agreed definition of the bioeconomy, there is substantial overlap – or a “common core” – in how the term is used. A widely cited formulation describes it as the “range of economic activities based on the utilization of biological processes and renewable resources for agricultural and industrial purposes” (German Bioeconomy Council 2018). Within this understanding, scholars have typically emphasized four related transformation dynamics: substituting fossil resources with bio-based raw materials, increasing productivity in bio-based primary sectors, improving efficiency in biomass use, and creating value through biological principles and processes beyond large-scale biomass production (Dietz et al. 2018). More recently, particularly in Latin America and parts of Africa, biodiversity-oriented bioeconomy approaches have also gained prominence (Förster et al. 2021; Aparicio 2022; Bastos Lima and Palme 2022; Queiroz-Stein et al. 2024). While these transformations aim to create economic opportunities based on a more sustainable resource base, they do not per se exclude unsustainable practices. They may also generate new forms of systemic stress and vulnerabilities.

From a social-ecological resilience perspective, the bioeconomy rests on bio-based production systems (BBPS), i.e., coupled social-ecological systems that use biological processes and ecosystem services to create economically valuable products and services (ge et al. 2016). The long-term development of the bio-economy depends on the resilience of these systems, i.e., their ability to respond to shocks and stresses while maintaining their functions at a desirable level (Holling 1973; Folke et al. 2010; Folke et al. 2016; Meuwissen et al. 2019; Feindt et al. 2020). Shocks such as extreme weather events, financial crises, technological breakthroughs and geopolitical conflicts, and long-term stresses such as increasing resource competition, climate change, ecosystem degradation and changing consumer preferences create resilience challenges for BBPS (Ge et al. 2016; Feindt et al. 2020: 636; Folke et al. 2021; Pörtner et al. 2023). If such challenges accumulate, unilateral action, market coordination or voluntary cooperation alone might not be sufficient to safeguard BBPS functions, in particular if collective action problems (e.g., managing water tables) or market failures (e.g., moral hazard undermining insurance solutions) are involved. BBPS resilience then becomes a potential matter for public policy intervention. Importantly, resilience and sustainability of BBPS are complementary concepts: “Sustainability is the long-term coherence of a system with its ecological, social and economic environment. Resilience is the ability of a system to cope with stress and shocks. Unsustainable systems can be very resilient, and sustainable systems can be very vulnerable” (Meuwissen et al. 2020).

Given the proclaimed importance of the bioeconomy and the limited likelihood that emerging BBPS can address accumulating resilience challenges without public support, a clearer understanding of these challenges is essential for scholars and policymakers. It is therefore crucial to examine whether and how resilience challenges are acknowledged and addressed in strategic bioeconomy policies. While sustainability concerns in bioeconomy policies have been widely studied (Dietz et al. 2018; Boyer et al. 2022; Siegel et al. 2022; Dietz et al. 2023; Vogelpohl 2023), resilience-related aspects have only recently received attention (Varanini et al. 2024; Proestou et al. 2025; Warchold et al. 2025; Goritz et al. 2026) and remain underexplored (Virgolino and Holden 2025), including resilience challenges. To address this gap, we analyze the salience and composition of resilience challenges in bioeconomy policies. Drawing on recent resilience literature, we conduct a systematic content analysis of 78 national bioeconomy policy documents from 50 countries (2005–2020), mapping the text share devoted to resilience challenges, the specific shocks and stresses identified, the affected sectors and functions, and the policy instruments proposed in response.

Section 2 introduces the key conceptual steps for identifying resilience challenges, affected sectors and functions, and proposed resilience-enhancing policy solutions. Section 3 explains the methodological steps, before Section 4 presents the results. Section 5 concludes the paper with a brief summary of the findings, key implications, and an outlook for further research.

## Concepts and Hypotheses

The resilience concept is based in ecological thinking and was later expanded to analyze coupled social-ecological systems (Holling 1973; Walker et al. 2004). At the most general level, resilience is the capacity of a system to maintain its functions in the face of perturbations in its environment (Folke et al. 2010). Perturbations comprise short-term shocks and long-term stresses which can accumulate, pushing a system towards critical thresholds where it is no longer able to maintain key functions (Folke et al. 2016; Meuwissen et al. 2019). The resilience of social-ecological systems is conceptualized as a set of different capacities that enable systems to respond to perturbations. The distinction between absorptive, adaptive and transformative capacities is now widely established (Berkes 2023). Absorptive capacities enable a system to withstand shocks and stress while continuing its operations. Adaptive capacities enable a system to change some of its operations or elements in response to or in anticipation of resilience challenges, while transformative capacities allow a system that is no longer viable or desirable to undergo fundamental change, including its operational logic or paradigm (Walker et al. 2004; Folke et al. 2010; Folke et al. 2016). Much of the resilience literature aims to identify system characteristics that support resilience, e.g., diversity, openness, tightness of feedback, modularity and system reserves (Meuwissen et al. 2019).

In the context of bioeconomy development and policy, the resilience perspective guides attention to vulnerabilities and capacities of the BBPS which provide the goods and services at the foundation of the bioeconomy (ge et al. 2016). Observable bioeconomy transformations usually imply an expansion and intensification in the use of land, natural resources and ecological systems (although emerging approaches which emphasize sustainable use of local biodiversity for more just and equitable bioeconomy development indicate that different development paths are possible; see Aparicio 2022; Bastos Lima and Palme 2022; Queiroz-Stein et al. 2024). Particularly those parts of the bioeconomy that build on the production of raw materials from open terrestrial or aquatic ecosystems can become vulnerable if, for example, environmental and climate change, over-use and over-exploitation result in deteriorating eco-system functions, or if negative perceptions of BBPS (e.g., monocropping agriculture) lead to the loss of legitimacy and to societal backlash. But also social challenges, such as demographic change and lack of skilled labor, or economic challenges such as lack of capital or trade conflicts, can test BBPS resilience. Public policies can contribute to an enabling environment (Mathijs et al. 2022) that strengthens resilience capacities or compensates for their lack in case of a crisis. Anticipation and recognition of resilience challenges is a precondition for effective policy responses (Feindt et al. 2020; Soriano et al. 2023).

It is therefore important to analyze whether and how resilience challenges are addressed in bioeconomy policies. Following established analytical categories in the resilience literature (Carpenter et al. Herrera 2017; Meuwissen et al. 2001; 2019), we distinguish statements on four dimensions of resilience:

1. Statements about economic, social, environmental, and political shocks or stresses that could threaten the desired functions of the bioeconomy and its production systems in the future (challenges: resilience *to what?*). While short-term stresses refer to potential future shocks that could affect the system, such as extreme weather or war events, long-term stresses refer to negative trends or worsening problems, such as climatic or demographic change.
2. Statements about BBPS (e.g., cropland or aquaculture), specific resources (e.g., corn or seaweed) or sectors (e.g., the agricultural sector) that are potentially affected by resilience challenges (object: resilience *of what?*). Considering the time-dependence of resilience (Carpenter et al. 2001), it is important whether statements address resilience challenges that affect the current bioeconomy, the development of the bioeconomy, or a future fully developed bioeconomy.
3. Statements about uses of bio-based resources (e.g., energy production from maize) or functions of BBPS, the bioeconomy or sub-sectors (e.g., generation of renewable energy, income and employment) that are potentially affected by resilience challenges (resilience *for what* purpose?).
4. Statements about instruments to address the resilience challenges (remedies: *what enhances* resilience?).

To guide our analysis, we formulate six hypotheses, based on previous comparative research on bioeconomy policies.

Salience: Despite the importance of the resilience of BBPS to the bioeconomy, bioeconomy policies have been mostly assessed as not sufficiently incorporating sustainability concerns, foregrounding the opportunities of bioeconomy transformations while omitting the challenges (Pfau et al. 2014; Vivien et al. 2019; Boyer et al. 2022). We therefore expect low salience of resilience challenges in bioeconomy policy documents (H1).

Time orientation: Resilience challenges can affect the bioeconomy at different time scales – from the presence to the distant future. Various factors are likely to lead to a focus on more immediate concerns, including psychological dispositions (time preference), availability heuristics and short-term political cycles (Laibson 1997; Frederick et al. 2002). We therefore expect that bioeconomy policy documents address significantly more short-term than long-term threats (H2).

Types of resilience challenges: Resilience to environmental stresses such as changing climate and water regimes are central to the functioning of BBPS. Stable and sufficient biomass production is the precondition for replacing fossil-based resources and creating reliable economic growth and jobs. Developments that could threaten this production should therefore be of critical concern (Staffas et al. 2013; Scarlat et al. 2015; Murray et al. 2017). Although more recent scholarship highlights socio-biodiversity-oriented bioeconomy approaches (Queiroz-Stein et al. 2024), earlier analyses of bioeconomy discourses during our study period consistently identified a strong economic focus (Bugge et al. 2016; Hausknost et al. 2017; Vivien et al. 2019) and the neglect of issues of social inclusion (e.g., smallholder participation) and societal dialogue (e.g., local councils) (Gerhardt et al. 2022). We therefore expect that bioeconomy policy documents mainly address economic and environmental challenges (H3).

Sectors: Established governmental visions of the bioeconomy have typically focused on the production of bioresources and bioenergy for industrial purposes, or on the development of a bio-based high-tech sector (Dietz et al. 2018; Proestou et al. 2024; Johnson et al. 2025; Lhuillery et al. 2025). We therefore expect that bioeconomy policy documents link resilience challenges mostly to these sectors, along with the broader agricultural and forestry sectors which provide most bioresources (H4).

Purposes: Distinct from the type of challenges addressed, this dimension focuses on the purposes or system functions that resilience is meant to safeguard (i.e., resilience for what). Different visions for the bioeconomy articulate different purposes: The biotechnology vision prioritizes economic growth and employment creation; the bioresources vision aims to achieve environmental sustainability while generating economic growth through bio-based production; only the bioecology vision prioritizes environmental sustainability, while economic growth and employment creation are secondary (Bugge et al. 2016; Vivien et al. 2019; Proestou et al. 2024). We therefore expect that economic purposes will be significantly more salient than environmental and social ones (H5).

Remedies: The biotechnology and bioresource visions are connected to public policies and private initiatives which aim at or imply the expansion of bioeconomy activities, while the bioecology vision connects biodiversity, conservation, and circular economy principles to include strong sustainability principles in the bioeconomy (Meyer 2017; Vivien et al. 2019). However, none of these visions strongly endorses far-reaching regulatory interventions (Goritz et al. 2025). Comparative research shows that only few national bioeconomy strategies explicitly address possibly negative consequences of bio-based transformations, and even sustainability-oriented strategies tend to rely primarily on soft policy approaches rather than binding regulatory measures (Dietz et al. 2018). We therefore expect a dominance of financial and informational support instruments over regulatory or procedural instruments (H6).

The next section explains the operationalization of these dimensions for the content analysis of bioeconomy policy documents.

## Methods and Data

**Fig. 1:**
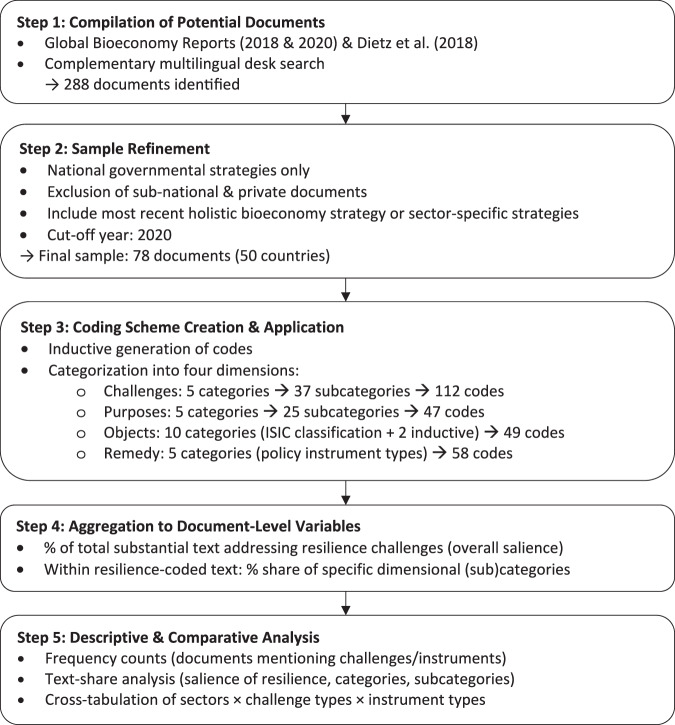
Flow chart of key analytical steps

**Fig. 2:**
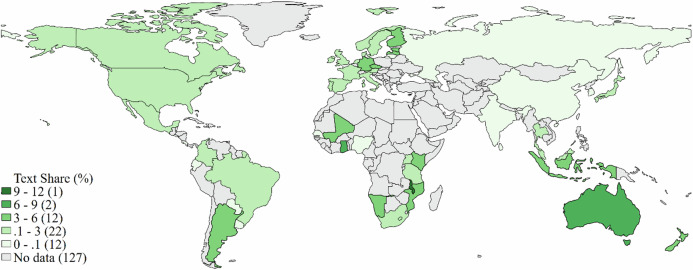
Geographical coverage of coded bio-related documents and average resilience text shares by country

*Note:* The average resilience text share is the unweighted arithmetic mean across a country’s distinct documents.

To analyze the salience and content of statements about resilience challenges in bioeconomy-related policy documents we conducted a qualitative-quantitative content analysis of 78 policy documents from 50 countries (mapped in Fig. 2). Coded text-segments were aggregated into document-level variables that capture the four resilience dimensions (object, purposes, challenges and remedies) and patterns identified. This section explains the document selection criteria and specifies the operationalization of the key concepts, summarized and illustrated in Fig. 1.

### Case Selection

To enable an analysis of those documents that most directly and comprehensively capture a country’s current bioeconomy orientation, an inventory of all bioeconomy policy documents was created. Drawing on the comprehensive and authoritative lists compiled by the 2018 and 2020 Global Bioeconomy Reports (IACGB 2020) and Dietz et al. (2018) – which together accounted for 223 of the identified documents – and complementing these with further desk research that yielded an additional 65 texts, 288 potentially relevant bioeconomy policy documents were identified. For the Google search, the following search term were used:

“(policy | strategy | plan | política | estrategia | politique | stratégie) and (bioeconomy | bio-economy | biotechnology | biomass | biofuel | bioenergy | bioindustry | circular | bioeconomía | biotecnología | biomasa | biocombustible | bioenergía | bioéconomie | biotechnologie | biomasse | biocarburant | bioénergie | circulair).”

We subsequently looked at the first 200 results, looking for direct references to national governmental bioeconomy or bioeconomy-related strategies, i.e. strategies promulgated by a national government, ministry, or ministry-linked agency. All strategies authored by sub-national governments (e.g., the regions of Flanders in Belgium) or private actors were excluded, as our analytical focus was on each country’s overarching national bioeconomy vision and its framing of resilience challenges at the highest governmental level. We acknowledge, however, that regional strategies can be more specific and operational. Their exclusion may therefore underrepresent more detailed or implementation-oriented approaches to resilience. Future research could complement our analysis by examining sub-national strategies to assess whether and how resilience is addressed differently across governance levels.

Since many of these documents did not represent the most direct or recent characterization of a country’s current bioeconomy policy, this list was further reduced in two steps. First, 16 countries had a holistic bioeconomy strategy. In these cases, the most recent version was included since holistic bioeconomy strategies provide the most direct, comprehensive and effective representation of a country’s prevailing bioeconomy policy doctrine. Second, for countries that had not published a holistic bioeconomy strategy, all bioeconomy-related documents were included. Documents were considered bioeconomy-related if they focused directly on at least one of the main areas of the bioeconomy, i.e. biotechnology, biomass, biofuels, biorefineries, bioindustries, bioenergy, the blue economy, or circular economy. If a country had several strategies per area (e.g. two biofuel strategies), only the most recent one was selected.

The final sample included 78 bioeconomy policy documents from 50 countries, illustrated in Fig. 2 and summarized in Appendix 1 (which includes document IDs used to reference documents in the results section.) As detailed in Appendix 2, the majority of policy documents are from high- or upper-middle-income countries (58); only three of the 16 holistic bioeconomy strategies were produced by governments outside the OECD. Biotechnology (19) and bioenergy (16) policies were the other most frequent types of documents in the sample. More than three-quarters (61) of the sample documents were published after 2010, and exactly half (39) after 2015. The sampling cut-off year of 2020 was set to coincide with the publication of the 2020 Global Bioeconomy Report, which, at the time of data collection, represented the most comprehensive and authoritative international overview of bioeconomy strategies. Using this report as a benchmark allowed for a systematic and consistent identification of relevant national documents. Data collection and coding were finalized before the release of the 2024 update. To preserve comparability and consistency within the sample, we excluded documents issued after 2020. Several major national strategies adopted since then (e.g., China’s 14th Five-Year Plan for Bioeconomy Development 2021–2025; India’s BioE3 policy; or Brazil’s 2024 bioeconomy strategy) therefore fall outside our analytical window. As illustrated in Appendix 2, overall patterns in challenge salience showed little variation over time within the study period, providing some indication that the core patterns identified here may remain relevant beyond 2020.

### Operationalization of Resilience Challenges

The analysis required to create variables that capture all relevant statements about the four dimensions of resilience-related statements (object, purposes, challenges, remedies). This was achieved in several steps. First, manual content analysis (Neuendorf 2017; Krippendorff 2019; Mayring 2022) was applied to the full text corpus to inductively generate a comprehensive list of codes potentially relevant to the four dimensions

These initial codes were then categorized and combined into a structured coding scheme (see Appendix 3). At the highest level, this coding scheme was divided into the four main dimensions (object, purposes, challenges, remedies). Each of these four dimensions was further disaggregated, using related and distinct coding systems. The 112 different, inductively identified, r*esilience challenges* were grouped into five distinct categories – ‘Economic’, ‘Environmental’, ‘Social’, ‘Political’ and ‘Research, Innovation & Technology’. Each of these categories was further disaggregated into up to thirteen distinct subcategories, which are detailed in the results section. In rare cases, codes and subcategories could be assigned to more than one category. For example, the subcategory ‘employment’ was coded as relating to both the ‘economic’ and ‘social’ categories. A nearly identical categorization system was used for the 47 different, inductively identified, *purposes*.

Regarding the *object* of resilience, the inductive analysis created 49 codes for different types of BBPS, products, resources, and sectors. These were classified according to the sectoral classification scheme of the fourth revision of the United Nations’ *International Standard Industrial Classification of All Economic Activities* (ISIC) (UNDESA 2008). Three codes – ‘bioeconomy at large’ (generic references to bioeconomy rather than to specific sub-sectors), ‘bioenergy’ and ‘biotechnology’ – were not well-aligned with existing ISIC categories and were added as new categories at the section level of aggregation (i.e. the highest level possible).

In addition, we distinguished whether resilience challenges referred to (i) currently existing bioeconomy structures and functions, (ii) bioeconomy elements that are actively being developed but not yet fully established, or (iii) planned or envisioned future functions of the bioeconomy. This distinction captures different stages in the development of the bioeconomy rather than specific chronological time horizons.

Regarding *remedies*, the inductive analysis generated 58 codes for statements about instruments proposed in the policy documents to address resilience challenges. Following established typologies of policy instruments (Hood and Margetts 2007; Böcher 2012), these were categorized as informational, cooperative, administrative, economic, or regulatory, according to their basic mode of functioning. The likelihood of detecting instruments related to the coded challenge depends on the distance between the two. Some documents list instruments close to the challenges, others contain separate sections, sometimes pages away from the challenge. Instruments were coded only if they were clearly related to statements about challenges. Thus, instruments mentioned without an explicit reference to a specific challenge may not have been coded, even if they were implicitly intended to address one. We consider this risk limited and preferable to introducing subjective interpretations by coding implicit or assumed linkages. Using this scheme, all documents were systematically coded using the content analysis software MAXQDA.

In a third step, codes were aggregated to create

- variables that represent the share of a document’s text that addresses resilience challenges as a percentage of the document’s total substantial text. These variables indicate the extent to which resilience challenges are generally salient in bioeconomy policy documents;
- variables which indicate the share of text with specific codes and categories related to objects, challenges, purposes and remedies within the document’s text share that was coded as addressing a resilience challenge. This allowed to identify patterns of specific content statements related to resilience challenges.^1^ Descriptive statistics were used to analyze the findings.

## Results

### Salience and Time-Orientation of Resilience Challenges

**Fig. 3:**
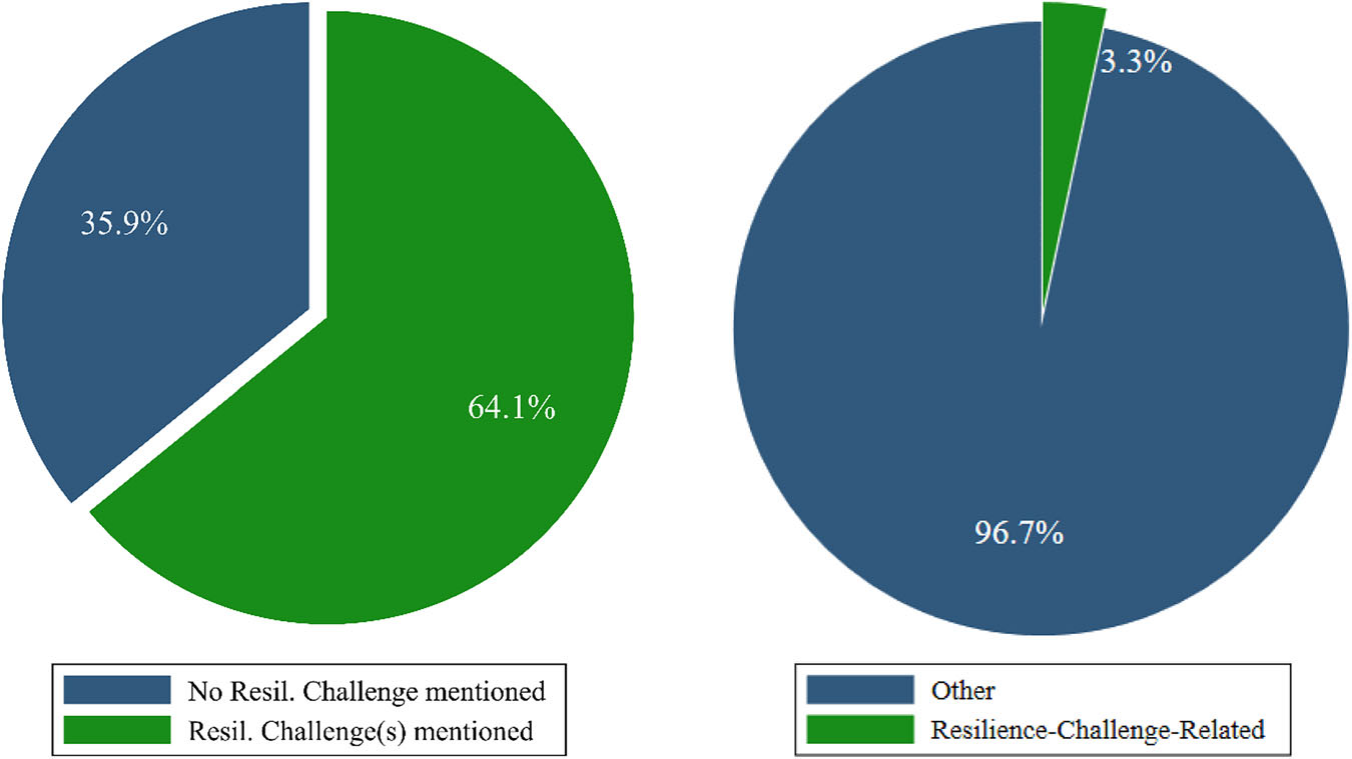
(Left) Share of documents that problematize resilience challenges; and (right) text share of resilience challenges in bioeconomy policy documents with mentioning of resilience challenges

Almost two-thirds (64.1%) of the 78 policy documents in our text corpus mention at least one resilience challenge (see Fig. 3, left side). Within these 50 documents (see Fig. 3, right side), on average 3.3% of the substantial text area discusses potential resilience challenges (the percentage across all 78 documents would be 2.1%). By comparison, the average amount of space devoted to discussing the main objectives of a strategy is 38% of relevant text. Existing barriers, such as a cumbersome regulatory system, that need to be overcome to develop the bioeconomy, take up over 5% of the average strategy.^2^ This suggests a rather low salience of resilience challenges in bioeconomy policy documents, generally confirming hypothesis H1.

However, in the 50 bioeconomy documents which mention such challenges, we found on average eight different resilience challenges. At the top were Indonesia’s “Master Strategy for Agricultural Development 2015–2045”^3^ (IDN14) and New Zealand’s “Primary Sector Science Roadmap” (NZL17), which each mention 29 distinct resilience challenges, followed by Italy’s bioeconomy strategy with 25 (ITA19). This indicates that at least a subset of the text corpus defied the low expectations. While this is not sufficient to reject hypothesis H1, it shows that ecological fallacy must be avoided. A detailed list of the proportion of text devoted to resilience challenges in all bioeconomy documents, and the number of different challenges mentioned, can be found in Appendix 1. Appendix 2 shows these two statistics across different country income groups, document types, continents, and years, without any discernible patterns. As expected, documents which mention more distinct challenges tend to have higher text shares dedicated to resilience challenges (the two variables correlate at 0.62). Some strategies deviate from this trend. For example, the Italian Bioeconomy Strategy, while mentioning the third-most distinct challenges, has a resilience challenge text share below average at 2.4%, because the long strategy lists many challenges in bullet points without discussing them in detail.

Regarding temporal orientation, most resilience challenges discussed in bioeconomy policies were negative trends (see Appendix 4). 96% of the text shares related to resilience challenges referred to long-term stresses, while only 11% referred to shocks which could threaten the functioning of the bioeconomy. This contradicts the expectation that bioeconomy policy documents address significantly more short-term than long-term threats and suggests that hypothesis H2 should be rejected.

**Fig. 4:**
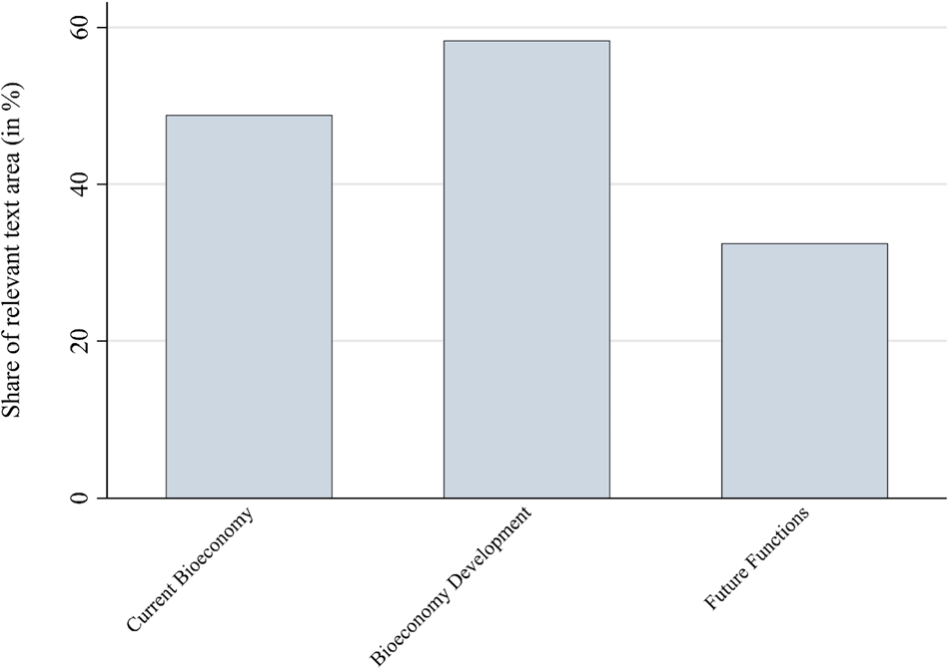
Challenge-related text share by time dimension (in %)

Another perspective on the temporal orientation is to analyze whether mentioned resilience challenges refer to the current bioeconomy, its development or its future. Most resilience challenges mentioned in the text corpus relate to the *development* of the bioeconomy (58% of the text) and the currently *existing* bioeconomy (49%), rather than the *future* functions of the bioeconomy to be established (32%) (see Fig. 4). This finding partly confirms hypothesis H2.

### Types of Resilience Challenges

**Fig. 5:**
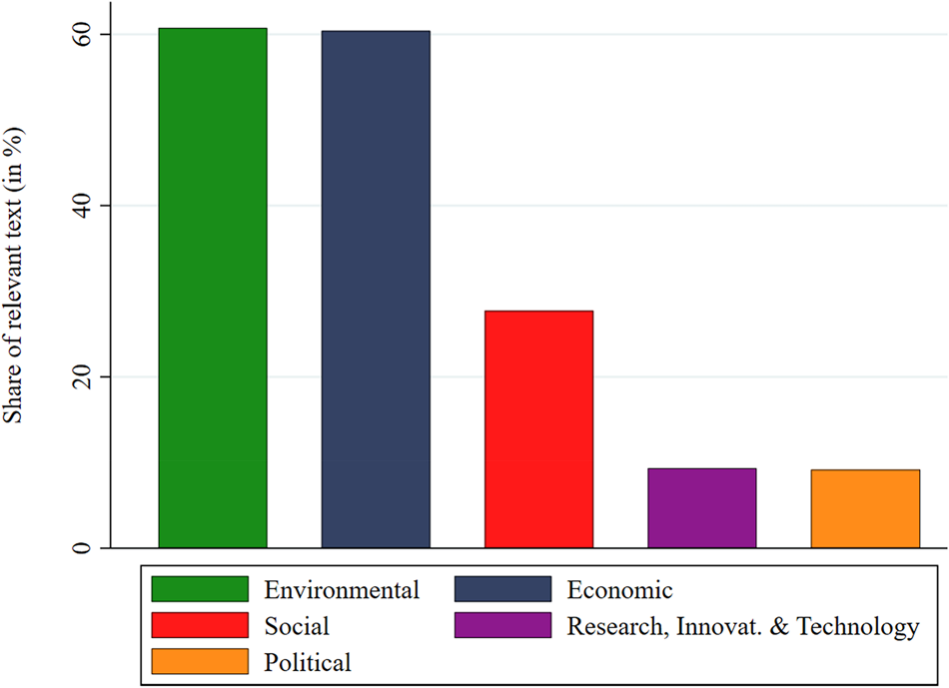
Challenge-related text share by categories (in %)

Figure 5 shows the proportion of resilience-challenge-related text by major category. As expected, environmental and economic threats to the bioeconomy were dominant, with about 60% of statements about resilience challenges referring to each category. However, social resilience challenges, while less salient, were still mentioned in nearly 28% of challenge-related text. Resilience concerns related to policy and to research, innovation, and technology, respectively, were each mentioned in about 9% of challenge-related text. Overall, these findings confirm hypothesis H3.

**Fig. 6:**
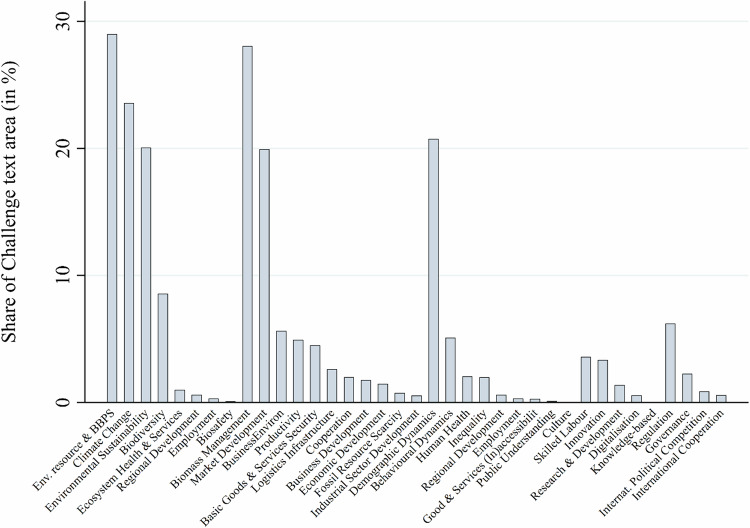
Sub-categories sorted by category and challenge text share

Figure 6 shows the findings for the more specific subcategories. Within the environmental category, challenges related to environmental resource concerns, climate change, and environmental sustainability were particularly prevalent. Regarding environmental resources, a particularly salient concern was competition for land, as land used for ecosystem services could, for example, be diverted to biomass production. In the medium and long term, the loss of ecosystem services could disrupt BBPS (e.g., habitat loss of pollinators needed for production plants), thereby threatening the desired functions of the bioeconomy. These and related environmental concerns were explicitly articulated, for example, in New Zealand’s 2017 Primary Sector Science Roadmap (NZL17), which states: “Changes in land and water use and in production systems, coupled with population growth and urban development, will continue to present significant environmental issues such as nutrient pollution, poor water quality, pests and diseases, and changed habitats including those important for our wild fisheries.” Direct climatic threats to BBPS from climate change were another key concern in many documents. For example, Latvia’s 2017 Bioeconomy Strategy (LVA17) states: “Economic activity of a human being causes significant climate change, reduces availability of fresh water, deteriorates quality of air and water, depletes ecosystems”, and these “risks (…) affect both agriculture and forestry.” The Latvian and New Zealand statements also mention unsustainable resource management as a challenge for the emerging bioeconomy.

In the economic category, challenges related to biomass management and market development were most salient. Biomass management was mainly linked to challenges from land competition – in particular, competition between biomass production for food or other bio-based products such as fuel. Another frequent concern was the availability of sufficient volumes of biomass to feed the bioeconomy transformation in the medium and long term. For example, the “Strategic Biomass Vision of the Netherlands towards 2030” (NLD16) asked whether “there is enough biomass worldwide to meet all needs”, adding that “the large-scale use of biomass for non-food applications may jeopardize food supply”, while also acknowledging that water usage for biomass production might “be amplified by climate change”.

The second major economic subcategory was market development. Here, several documents articulated concerns about increasing international competition. For example, Estonia’s Development Plan for Enhancing Bioenergy (EST07) raised the potential threat of “more rapid development of competing markets” due to their “considerably greater technological and financial potential”, while New Zealand’s Primary Sector Roadmap (NZL17) and Italy’s holistic bioeconomy strategy (ITA19) were concerned about a potential future “loss of our markets to competitors” and “increasing competition at worldwide levels”.

Demographic dynamics was the third most salient subcategory, coded in over 20% of the challenge-related text segments. While mainly associated with the social category, it was often closely related to environmental and economic resource concerns. In low- and middle-income countries (e.g., UGA08 and ZAF13), high rates of population growth were a worry, linked to increasing pressure on environmental resources and eco-systems and to increasing demand for food, thereby exacerbating problems of land and biomass availability. As one example of many, Malawi’s Biomass Energy Policy articulated concerns that the “continuing increase in population (…) will place intolerable strains on the land and woody resource base in certain areas” (Government of Malawi 2009).

While texts from high-income countries mentioned rapid population growth rates in other parts of the world (e.g. FIN18 and DEU20), the concern for their own countries was rather population decline in rural areas. Particularly in documents from European countries, where bioeconomy strategies tend to follow bio-resource visions (Hausknost et al. Meyer 2017; Vogelpohl and 2017; Töller 2021), such demographic trends were seen as potentially limiting the production capacity for biomass and as a problem for the overarching goal of rural development. For example, the Italian bioeconomy strategy (ITA19) problematized “dramatic changes in land use” due to land abandonment in the countryside, which could lead to a significant “overall decrease in agriculture, grasslands and semi-natural habitats” in the future.

Among the two less frequent categories – research, innovation, and technology, and policy – challenges related to regulation and skilled labor were most frequent. A frequent concern was that regulation might not keep pace with developments in the bioeconomy or that future administrations would change regulations to the detriment of the bioeconomy (e.g., in AUS14, COL11, GHA19, ITA20, KEN06, MOZ12, UGA08). Skilled labor, on the other hand, was presented as a critical factor, especially for the more technology-intensive parts of the bioeconomy. Several documents articulated this as a critical existing or future constraint on the development of the bioeconomy (COL18, JPN19, ZAF13, or URY12 being among the most explicit).

### Sectors of Concern

**Fig. 7:**
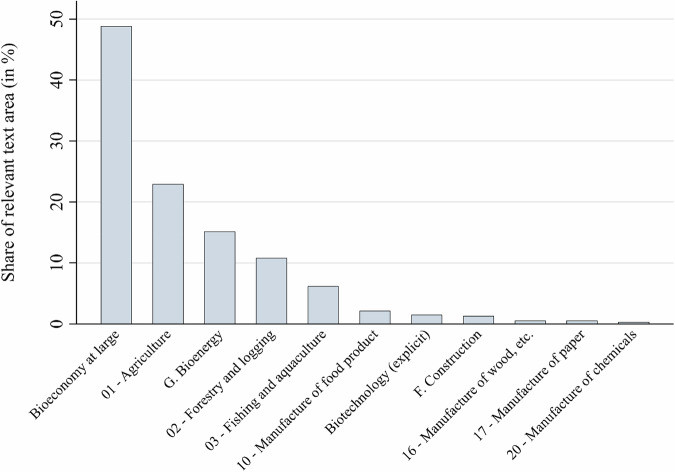
Sectors affected by resilience challenges. Note: The number and letter prefixes for some of the sectors are derived from their respective ISIC section- and division-level equivalents

Figure 7 shows which sectors were linked to a resilience challenge. It comprises the ISIC section- and division-levels for a more parsimonious visualization. Almost half (48.9%) of the resilience-coded text shares referred to the bioeconomy at large as being challenged, rather than specifying a particular sector. Among the explicitly mentioned sectors, agriculture (23%) and bioenergy (15%) were the most salient. Forestry (11%) and fishing & aquaculture (6%) were also relevant. Biotechnology as a specific sector was rarely linked to resilience challenges (at 1.5%), and less than 4% of the resilience-coded text was related to manufacturing as a sector. The findings confirm the expectation that resilience concerns are mostly linked to sectors which support the dominant vision of the bioeconomy as the production of bioresources and bioenergy, with agriculture, forestry and fisheries as the main sources of supply. The bio-based high-tech sector, however, is rarely mentioned in connection with resilience concerns. Hypothesis H4 is therefore only partially confirmed.

**Fig. 8:**
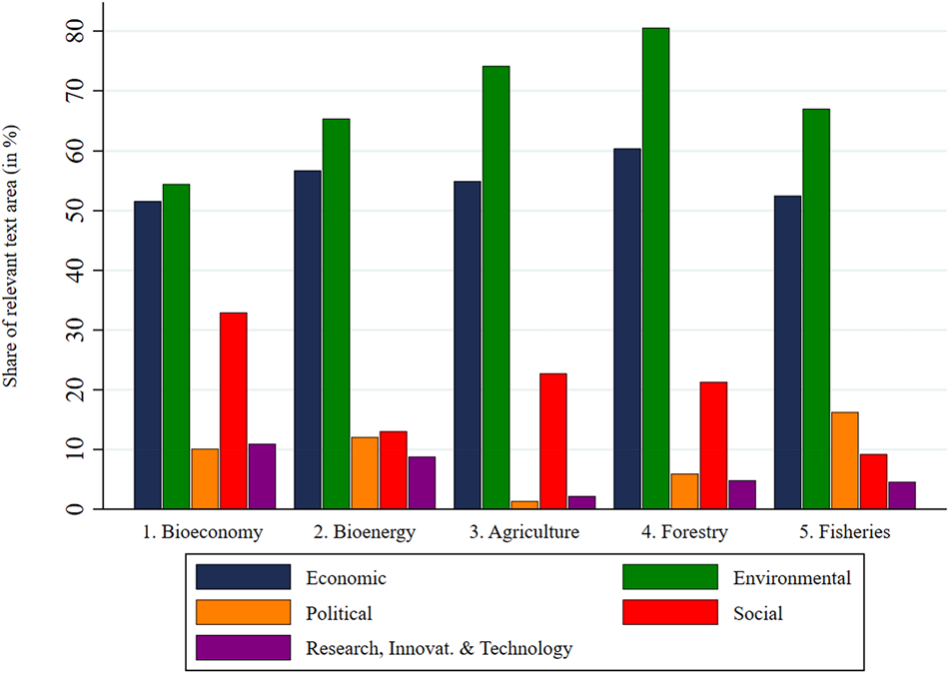
Degree to which the most affected sectors were linked to the distinct resilience challenge categories

Figure 8 analyses to which types of resilience challenges the most affected sectors were linked in the documents. For all five sectors, environmental challenges were most salient, followed by economic challenges. Environmental concerns were most salient for forestry and agriculture, with more than 75% of resilience-coded text share. Economic challenges ranged between 52% and 60% for the five sectors. Social challenges were by far most salient for the bioeconomy at large, followed by agriculture and forestry. Research, innovation & technology challenges were mostly linked to the bioeconomy at large and to bioenergy. Political challenges were most salient for the fisheries and aquaculture sector and almost absent for the agricultural sector.

### Purposes and Functions

**Fig. 9:**
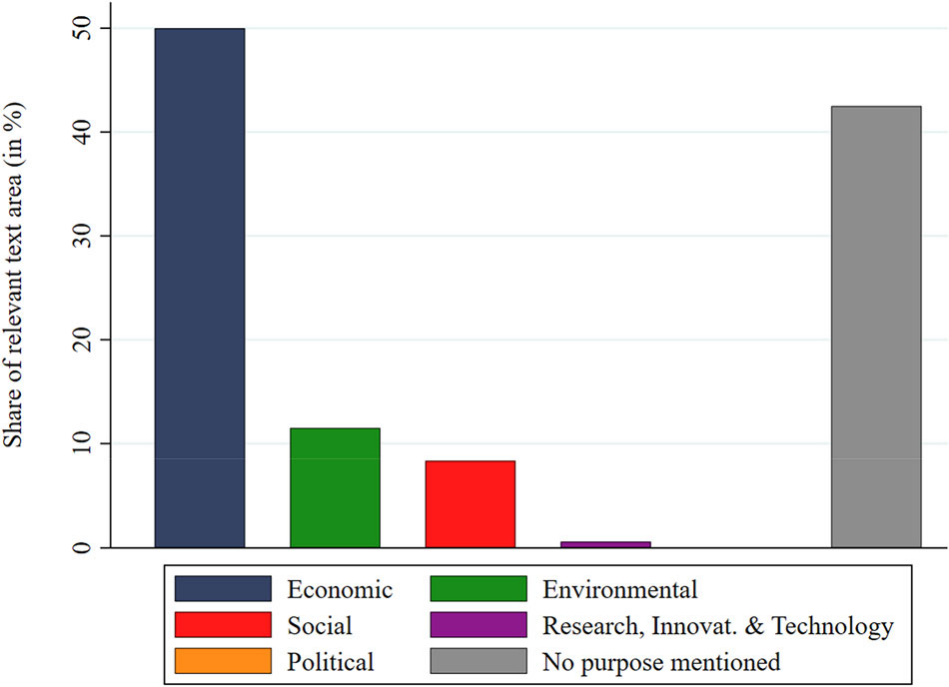
Share of resilience-coded text mentioning purpose or function of challenged sectors. Note: The share of the category ‘political’ was zero

Figure 9 shows the share of resilience-coded text that mentions different purposes and functions of the potentially challenged sectors. An astonishing 42.5% of the resilience-coded text do not mention any purpose or function of a sector. Among the remaining 57.5%, the economic category is dominant with a text share of 50%, followed by the environmental (11.5%) and social (8.4%) categories. Research, innovation & technology and political purposes play little to no role at 0.6% and 0% respectively (both probably seen more as means rather than ends in themselves).

**Fig. 10:**
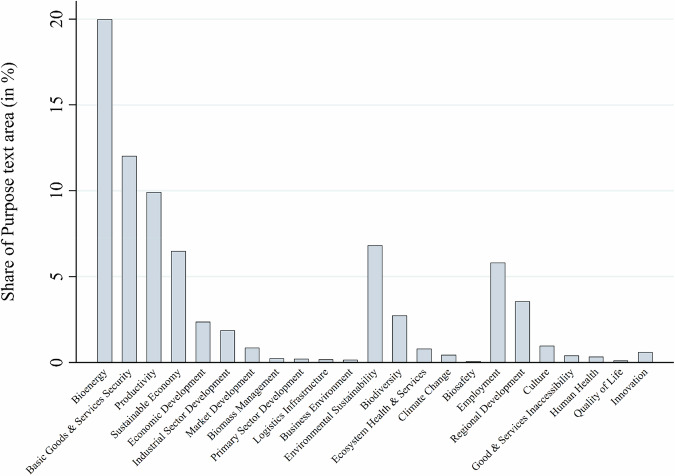
Purposes or functions linked to resilience concerns by sub-category

Figure 10 shows the most salient purpose/function subcategories. The dominance of economic purposes/functions is mainly driven by four subcategories: the provision of bioenergy (20% share of purpose-coded text), security of basic goods and services (12%), productivity (10%) and sustainable economy (6.5%). Environmental purposes were mostly notions of environmental sustainability at large (6.8%) and biodiversity (2.7%). Social purposes were mainly employment generation (5.8%) and regional development (3.5%). Overall, the findings show the expected dominance of economic purposes and strongly confirm hypothesis H5.

### Instruments as Remedies

**Fig. 11:**
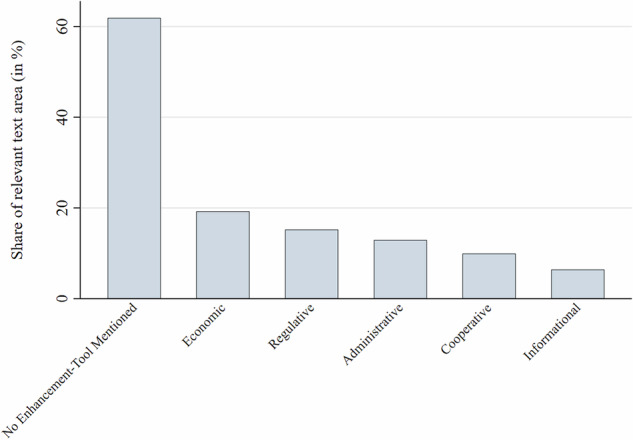
Relative importance of different instrument types to address resilience challenges mentioned

**Fig. 12:**
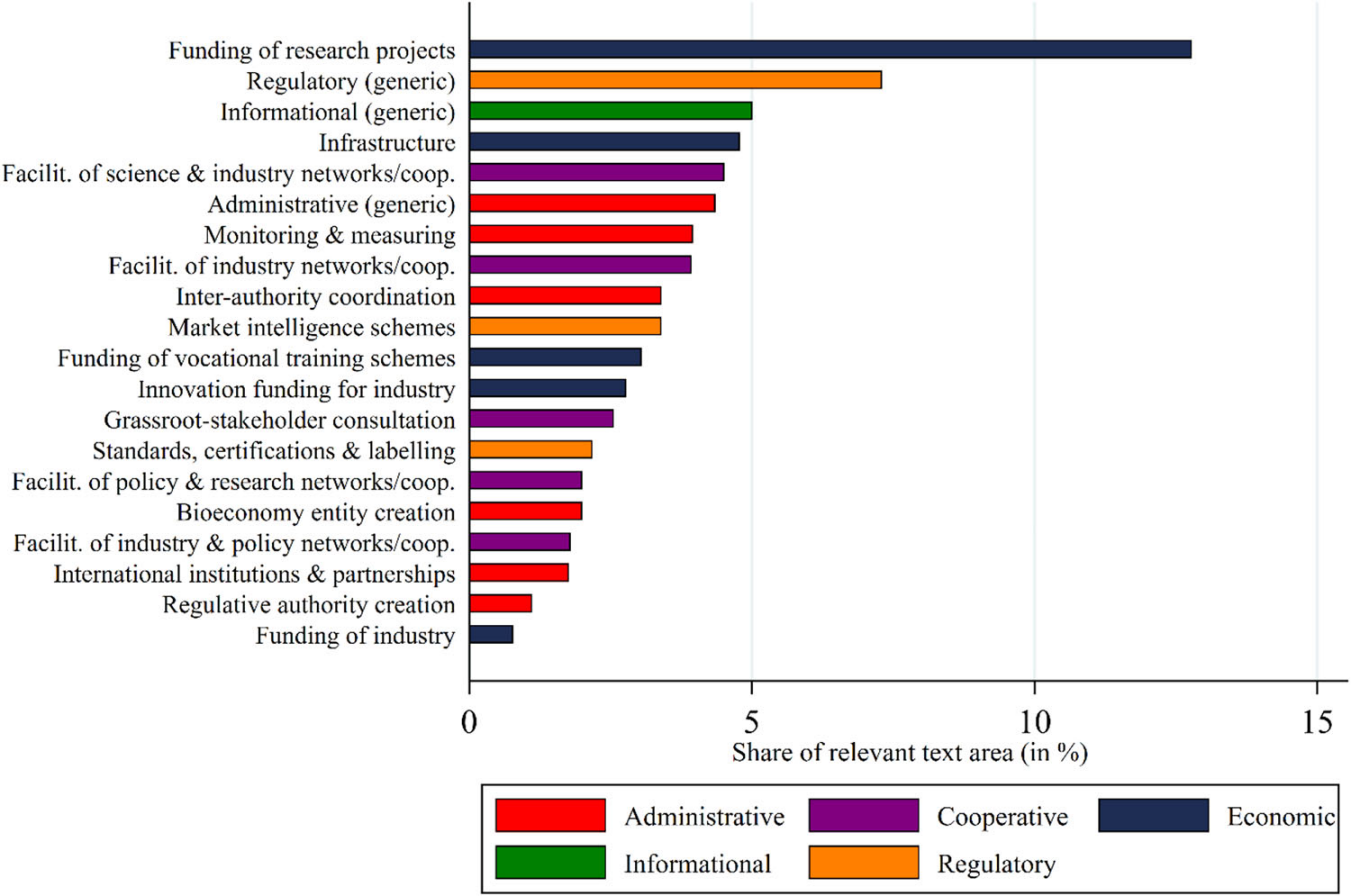
The 20 most relevant resilience-enhancing instruments mentioned

Figure 11 shows the share of bioeconomy documents which mentioned different types of policy instruments. 62% of the challenge-coded text did not mention any resilience-enhancing instruments, while 38% did. Economic instruments were mentioned in 19% of challenge-coded text. Most salient in this category were financial support measures (such as loans or subsidy schemes) for research institutions, industry, businesses and vocational training providers, and the establishment of relevant infrastructure. 15% of the challenge-coded text mentioned regulatory policy instruments, such as laws. Administrative tools (e.g., the creation of inter-authority coordination or greater monitoring and measuring capacities), cooperative tools (e.g., the facilitation of state-business-science networks), and informational tools (e.g., the dissemination of market intelligence or public media campaigns) were mentioned in 13%, 10%, and 6.4% of the relevant text shares respectively. Figure 12 details the 20 most salient instruments aimed at enhancing resilience. Three of the six most salient codes refer to generic instrument types (e.g., “regulation” broadly rather than a specific law), indicating low policy specificity.

The findings only partially confirm hypothesis H6. While financial support instruments were most salient as expected, informational instruments only played a minor role, even trailing behind administrative and cooperative tools, while regulatory instruments were almost as salient as financial tools.

**Fig. 13:**
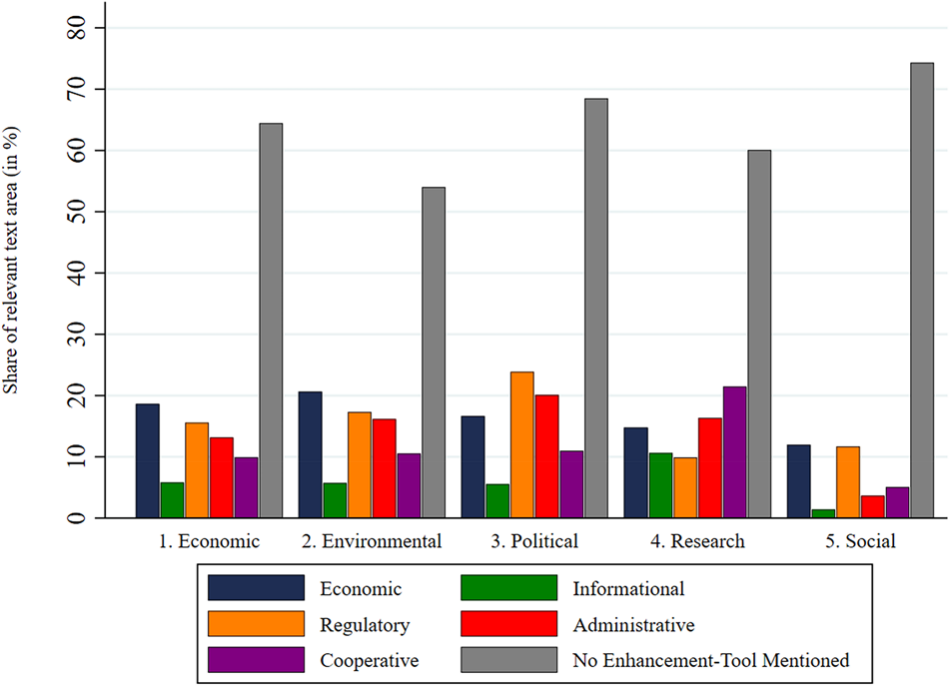
Policy instrument types proposed by resilience challenge category

A more detailed analysis shows differences in the salience of the instrument types linked to different types of resilience challenges. Figure 13 shows the text share linked to each instrument category as a percentage of the text share coded for each resilience challenge category. The share of challenge-coded text without any remedy ranged from 52% for environmental challenges to 76% for social challenges. Hence, environmental challenges were most likely to be linked to possible policy measures. Economic and regulatory instruments were the most frequently mentioned remedies for economic, environmental and social challenges. In contrast, policy challenges were mostly linked to regulatory and administrative instruments, and research and innovation challenges to informational and administrative tools. Hence, the dominance of economic tools in the text corpus is strongly driven by the strong salience of economic and environmental compared to other challenges.

## Discussion

The qualitative-quantitative content analysis of 78 bioeconomy policy documents from around the world found that a majority of these documents articulated often several of an overall broad range of challenges to the resilience of the bioeconomy and its sub-sectors. These challenges were often linked to specific purposes or functions at risk, but rarely to more or less specific policy instruments as remedies to address the challenges. The results therefore confirm the general relevance of the resilience concept for the analysis of strategic bioeconomy policy documents, as well as the absence of a full articulation of resilience thinking in most bioeconomy policies.

The results did confirm most, but not all hypotheses which were formulated based on previous analyses of bioeconomy strategies and visions (for an overview, see Table 1). In line with hypothesis H1, the salience of resilience challenges was generally rather low, confirming earlier findings on the relative neglect of sustainability concerns and systemic long-term risks in bioeconomy policies (Vivien et al. 2019; Boyer et al. 2022; Warchold et al. 2025). H1 expected resilience challenges to occupy only a marginal share of strategic documents, and the very small average text shares (3.3%) clearly support this assumption. However, differences were significant. While one third of the documents did not mention any resilience challenges, others named large numbers of them or devoted significant text share to resilience issues, pointing to stronger resonance of resilience thinking in some places.

**Table 1:** Overview of hypotheses and their alignment with the data

Hypothesis	Related Figures	Empirical Assessment
*H1:* Low salience of resilience challenges in bioeconomy policy documents.	3	Supported
*H2:* More short-term than long-term threats.	4	Partially supported
*H3:* Prioritization of economic and environmental challenges.	5, 6	Supported
*H4:* Focus on bioresource (incl. agriculture and forestry), bioenergy, and biotechnology sectors.	7, 8	Partially supported
*H5:* Economic purposes are more salient than environmental and social ones.	9, 10	Supported
*H6:* Dominance of financial and informational support instruments over regulatory or procedural instruments.	11, 12, 13	Partially supported

The expected short-term orientation (hypothesis H2) manifested only partly in the results. While H2 anticipated a stronger emphasis on short-term shocks, resilience challenges, when mentioned, mostly referred to long-term stresses. At the same time, resilience challenges were mostly related to the developing or the currently existing bioeconomy (partially aligning with H2), while one third of the challenges were related to future functions of the bioeconomy. These findings point to possible openings for a more anticipatory approach to resilience issues at least in some countries. In contrast, neglect of serious long-term threats can lead to insufficient preparation. An example is Malaysia, which, according to climatologists, could likely lose large swathes of its palm oil plantations due to climate change within a few decades (Sarkar et al. 2020). Given that the crop and its biomass constitute a central pillar of the country’s bioeconomy, the absence of serious risk considerations in strategic policy documents points to a lack of resilience capacities such as anticipation and preparedness.

In line with hypothesis H3, when resilience challenges were identified, these were predominantly environmental and economic stresses. This reflects a certain awareness of environmental risks, in particular from changing climate and water regimes, for biomass production (Scarlat et al. 2015; Murray et al. 2017; Warchold et al. 2025). It also betrays uncertainty about the competitiveness of the emerging bioeconomies against other sectors and nations, with a growing number of countries building significant expertise and infrastructure in biotechnology (e.g., biorefineries) or in the production of specific bio-based products (e.g., biopharmaceuticals). The prominence of economic challenges in our data aligns with previous diagnoses of a strong economic focus in bioeconomy discourses (Bugge et al. 2016; Hausknost et al. 2017; Vivien et al. 2019; Proestou et al. 2024). The strong salience of concerns about biomass management and land competition resonates with long-standing academic and political debates about ‘fuel versus food’ trade-offs (Thompson 2012), which have motivated policy shifts towards more closed material cycles and biotechnological innovations beyond biogas plants, e.g., cultured meat and precision fermentation. The relatively low salience of social challenges, among which demographic developments dominated over health, inequality and accessibility concerns, mirrors the results of earlier studies which found little attention in bioeconomy policies to issues of social inclusion (Gerhardt et al. 2022; Proestou et al. 2024). The strong salience of concerns about rural demographics echoes long-standing discussions in rural and development studies (Johr 2012). Concerns about unstable governmental support for the bioeconomy under changing political circumstances were validated in cases like the UK’s withdrawal of its 2018 bioeconomy strategy in 2021 or Colombia after the presidential election in 2022 (Varanini et al. 2024).

The relatively low salience of political challenges in the text corpus might reflect the comparatively calm geopolitical situation during the period of analysis. One might expect more consideration of strategic resource autonomy in more recent strategies, in particular those produced after Russia’s invasion of Ukraine and the second Trump presidency. The low salience of research, innovation and technology challenges resonates with observations that policymakers often hold very optimistic views of bioeconomy development (Backhouse et al. 2021; Eversberg et al. 2023). The most salient challenge in this category – the scarcity of skilled labor – matches established academic discussions (Bröring and Vanacker 2022; Ding and Grundmann 2022; Pascoli et al. 2022).

Resilience concerns were mostly linked to sectors which produce bioresources and bioenergy. Agriculture, forestry and fisheries have long enjoyed particular governmental support in most OECD countries and their producer interests clearly found a way into bioeconomy policies. Surprisingly, the bio-based high-tech sector was rarely mentioned in connection with resilience concerns (partial rejection of hypothesis H4). These findings could indicate either a dominant influence of the bioresource vision over the biotechnology and bioecology visions (Bugge et al. 2016; Proestou et al. 2024); or proponents of the biotechnology sector, while influential, might be less concerned about resilience challenges (Varanini et al. 2024). Meanwhile, the influence of bioecology visions might be best traced in the articulation of concerns about environmental resilience challenges. In any case, the prominence of the agriculture and bioenergy sectors resonates with the strong salience of biomass management and market development as major economic challenges and with salient social challenges such as declining rural populations and outdated or inadequate rural infrastructures.

As expected, when bioeconomy policies in the text corpus mentioned specific bioeconomy purposes or functions under threat, these were mostly economic ones, with environmental and social functions being much less salient (hypothesis H5). This finding resonates with observations that policymakers, while acknowledging the environmental dimension of the bioeconomy, prioritize its economic purposes and functions (Bugge et al. 2016; Hausknost et al. 2017; Vivien et al. 2019; Puder and Tittor 2023; Proestou et al. 2024; Varanini et al. 2024). The low salience of environmental purposes and functions in the text corpus contradicts narratives of the bioeconomy as a more ecological alternative to the fossil-based economy and hints to low influence of the bioecology vision.

The finding that most text shares about resilience challenges were not linked to any policy instrument suggests that the resilience concept has not yet been well elaborated in the analyzed bioeconomy policy documents. It might also indicate the generally abstract, programmatic and strategic character of many of these documents (Goritz et al. 2025). The fact that financial support and regulatory instruments were most salient when instruments were mentioned (only partly confirming hypothesis 6) corresponds to concerns about competitiveness and about environmental problems which call for strong steering mechanisms. The significant presence of administrative tools reflects observations that the governance framework for the bioeconomy is only emerging and requiring coordination across multiple policy sectors and levels of governance (Töller et al. 2021). Informational instruments, such as information campaigns and websites, as well as vocational training, which could help to address environmental and social concerns, were rarely mentioned. Cooperative instruments were over-proportionally mentioned to address research and innovation challenges. This matches frequent complaints in the bioeconomy discourse about insufficient collaboration between research and private sector towards commercialization along the value chain, especially in the research-intensive biotechnology sector (Woźniak and Twardowski 2018; Kampers et al. 2022).

## Conclusion

Bioeconomy transformations represent significant potential pathways to sustainable global development. Their success, however, depends on the resilience of bio-based production systems to a wide range of environmental, economic, social, and political challenges. This paper aimed to provide a better understanding of the extent to which governments recognize and address such resilience challenges in their bioeconomy policies. A systematic content analysis of 78 bioeconomy policy documents around the world mapped the salience, time-orientation and character of the articulated resilience challenges, the sectors and functions challenged, and the measures mentioned to increase resilience.

Overall, the study produced several key findings. First, while two-thirds of the 78 policy documents mentioned at least one resilience challenge, the average space devoted to their discussion was limited. While resilience challenges occupied on average only 3.3% of the text in documents that mentioned them (2.1% across the full sample), these documents nevertheless identified an average of eight distinct resilience challenges, with some strategies (e.g., Indonesia and New Zealand) articulating up to 29 distinct challenges. Second, environmental and economic stresses dominated the challenge-related text, each accounting for roughly 60% of coded challenge statements, whereas social challenges accounted for 28% and political and R&I challenges for about 9% each. Third, most resilience challenges referred to the current (49%) or developing (58%) state of the bioeconomy, though nearly one third (32%) of challenge-related text addressed planned or future bioeconomy functions. Fourth, while most resilience challenges were linked to the bioeconomy as a whole (48.9%), agriculture (23%) and bioenergy (15%) emerged as the most salient specifically affected sectors, followed by forestry and fisheries, indicating concerns about biomass production. The affected functions of these sectors were mostly described as economic and to a lesser extent as environmental purposes. Finally, nearly two-thirds of challenge-coded text did not mention any resilience-enhancing instrument, and when instruments were mentioned, economic (19%) and regulatory (15%) tools were most salient. Overall, the findings indicate a strong influence of the bioresource vision of the bioeconomy and resilience concerns that are strongly linked to established production sectors such as bioenergy, agriculture, and forestry. The biotechnology vision appeared to have few links to the articulation of resilience concerns in the 78 bioeconomy policy documents.

Compared with concerns about the long-term resilience of BBPS in the scientific literature, the findings raise the question whether resilience challenges have been sufficiently reflected and addressed in bioeconomy policies. While some countries reflected and addressed multiple and accumulating resilience challenges, others did not engage at all with this perspective. These findings suggest that resilience considerations remain unevenly institutionalized in bioeconomy policymaking. Strengthening anticipatory capacities, stakeholder dialogue, and explicit consideration of environmental risks could enhance the integration of resilience thinking into strategic bioeconomy governance.

Future research could build on this study in at least two ways. First, many of the analyzed strategies were published at early stages of the policy process and are characterized by rather general, agenda-setting statements rather than concrete policy guidance. Follow-up studies including later-stage policy instruments and implementation documents would therefore be valuable. Second, future research should examine how bioeconomy strategies are formulated and whose perspectives they reflect. Comparative analyses of policy processes and country contexts could help identify structural, political and cognitive factors that shape the recognition of resilience challenges, including patterns of actor inclusion, economic dominance, and differences in national risk exposure. Understanding these dynamics is essential for assessing whether bioeconomy transformations are being governed in ways that are not only economically ambitious, but also resilient in the face of accelerating environmental and societal change.

## Data Availability

The replication data can be found in the Harvard Dataverse at 10.7910/DVN/XULY9G.

## Appendix

Appendix 1: Selected Key Statistics Across Analyzed Bioeconomy Policy Documents

**Table:**

Country	Year	Docu-ment ID	Document Name	Distinct Challenges Mentioned (#)	Challenge Text Share (%)	Challenges					No Enhance-ment-Tool
						Econ-omic	Social	Environ-mental	Political	Research, et al.	
Argentina	2016	ARG16	Bioeconomía Argentina	3	3	62	0	100	0	0	82
Australia	2014	AUS14	Opportunities for Primary Industris in The Bioenergy Sector: National Research, Development and Extension Strategy Workplan	7	4	70	0	94	30	0	78
Australia	2018	AUS18	Biotechnology and agriculture in Australia: policy snapshot	6	13	74	0	74	26	0	26
Austria	2019	AUT19	Bioeconomy a strategy for Austria	10	3	79	0	39	6	0	88
Brazil	2016	BRA16	Estrategia Nacional de Ciencia, Technologia e Inovacao	10	4	25	56	59	0	0	100
Brazil	2018	BRA18	Plano de Ação em Ciência, Tecnologia e Inovação em Bioeconomia	2	1	0	100	0	0	0	100
Brazil	2020	BRA20	Plano Decenal de Expansão de Energia 2029	0	0						
Canada	2019	CAN19	Canada's Bioeconomy Strategy	5	1	77	73	57	0	0	23
China	2016	CHN16	13th FYP on Bioindustry Development	0	0						
China	2016	CHN16	13th FYP for Strategic Emerging Industries	0	0						
Colombia	2011	COL11	Política par el Desarrollo Comercial de la Biotecnología a partir del uso Sostenible de la Biodiversidad	0	0						
Colombia	2016	COL16	Colombia Bio	0	0						
Colombia	2018	COL18	Colombia Green Growth Roadmap	3	1	69	0	31	30	0	69
Costa Rica	2020	CRI20	National Bioeconomy Strategy – Costa Rica 2020 – 2030	0	0						
Czech Republic	2019	CZE19	Bioeconomy concept in the Czech Republic from the perspective of the Ministry of Agriculture 2019 – 2024	15	4	63	17	50	0	18	74
Denmark	2013	DNK13	Plan for Growth for Water, Bio & Environmental Solutions	1	4	100	0	0	0	0	100
Denmark	2014	DNK14	Denmark as growth hub for a sustainable bioeconomy	2	1	0	100	100	0	0	100
Denmark	2018	DNK18	Strategy for Circular Economy	2	2	45	0	55	0	0	45
Ecuador	2019	ECU19	Lineamientos Para El Fomento Del Bioemprendimiento	0	0						
Estonia	2007	EST07	Development Plan on the Promotion of Biomass and Bioenergy Use for 2007–2013	8	6	80	0	47	20	7	100
Finland	2014	FIN14	The Finnish Bioeconomy Strategy	7	2	100	56	36	0	0	67
Finland	2018	FIN18	Competitive Advantage from Clean Food and Responsible Bioeconomy and Circular Economy	20	8	37	42	33	25	0	71
France	2018	FRA18	A Bioeconomy strategy for France (Goals & Action Plan)	10	1	100	55	45	0	0	76
Germany	2020	DEU20	Nationale Bioökonomiestrategie	10	4	27	26	46	9	19	91
Ghana	2019	GHA19	Ghana renewable energy master plan	15	7	56	7	51	51	9	30
India	2015	IND15	National Biotechnology Development Strategy 2015 - 2020	1	0	100	0	100	0	0	0
India	2018	IND18	National Policy on Biofuel	0	0						
Indonesia	2014	IDN14	Bioenergy Policies and Regulation in Indonesia	0	0						
Indonesia	2014	IDN14	Master Strategy for Agricultural Development 2015 - 2045	29	6	47	30	62	7	8	76
Ireland	2018	IRL18	National Policy Statement on the Bioeconomy	3	1	100	0	100	100	0	0
Italy	2019	ITA19	BIT II - Bioeconomy in Italy: A new bioeconomy strategy for a sustainable Italy (including Action Plan)	25	2	44	22	80	27	19	63
Japan	2019	JPN19	Bio Strategy Japan 2019/2020	7	2	7	16	0	34	65	100
Kenya	2006	KEN06	A National Biotechnology Development Policy	8	4	68	19	100	0	0	100
Kenya	2008	KEN08	Strategy for Developing the Bio-diesel Industry in Kenya	2	3	32	0	100	0	0	32
Latvia	2017	LVA17	Latvian Bioeconomy Strategy 2030 (LI-BRA)	14	5	47	37	61	27	0	95
Lithuania	2010	LTU10	National Renewable Energy Action Plan	0	0						
Lithuania	2014	LTU14	Action Plans on Biomass Energy, Biomolecular Biotechnologies and Biorefineries promotion as part of the Lithuanian Smart Specialisation Programme	0	0						
Malawi	2009	MWI09	Malawi Biomass Energy Strategy	9	11	52	35	53	12	0	83
Malaysia	2005	MYS05	“National Biotechnology Policy” (2005-2020)	0	0						
Malaysia	2009	MYS09	Malaysia´s National Blue Ocean Shift	0	0						
Malaysia	2013	MYS13	National Biomass Strategy 2020: New wealth creation for Malaysia’s biomass industry (Version 2.0)	0	0						
Malaysia	2015	MYS15	Bioeconomy Corportion Bioeconomy Transformation Programme (BTP)	4	2	100	34	34	0	0	34
Mali	2008	MLI08	Stratégie Nationale pour le Développement des Biocarburants en Mali & Loi pour l'etabilissement de l'Agence Nationale de Développement des Biocarburants	6	3	100	72	72	0	0	0
Mauritius	2013	MUS13	The Ocean Economy	7	1	38	15	62	0	0	100
Mexico	2009	MEX09	Estrategia Intersecretarial de los Bioenergéticos	5	6	77	0	100	0	21	70
Mexico	2020	MEX20	Transition Strategy to Promote the Use of Cleaner Technologies and Fuels	0	0						
Mozambique	2009	MOZ09	Política e Estratégia de Biocombustiveis	3	1	100	0	100	0	0	0
Mozambique	2012	MOZ12	Mozambique Biomass Energy Strategy	12	8	73	58	90	17	5	64
Namibia	2014	NAM14	The National Programme on Research, Science, Technology & Innovation	15	3	44	10	45	17	27	50
Netherlands	2016	NLD16	Strategic Biomass Vision for the Netherlands towards 2030	6	3	85	0	44	15	0	100
Netherlands	2018	NLD18	The position of the bioeconomy in the Netherlands	0	0						
New Zealand	2017	NZL17	Primary Sector Science Roadmap - Te Ao Turoa	29	6	53	18	97	0	9	63
Nigeria	2007	NGA07	Nigeria Biofuels policy and incentives	0	0						
Nigeria	2015	NGA15	National Biotechnology Development Agency Bill	0	0						
Norway	2016	NOR16	Familiar resources – undreamt of possibilities. Government Bioeconomy Strategy	12	3	72	27	13	0	28	56
Paraguay	2011	PRY11	Politica y Programa Nacional de Biotecnoloía Agropecuaria y Forestal del Parauay (2011)	0	0						
Portugal	2013	PRT13	National Ocean Strategy 2013-2020	10	3	72	72	77	0	0	66
Portugal	2017	PRT17	National Plan for the Promotion of Biorefineries	3	1	45	0	100	0	0	45
Portugal	2017	PRT17	Circular Economy Action Plan	2	1	100	100	0	0	100	100
Portugal	2019	PRT19	Portuguese Bioeconomy Strategy Roadmap	0	0						
Russia	2012	RUS12	Comprehensive Program and Roadmap for the Development of Biotechnology in Russia by 2020	0	0						
Senegal	2007	SEN07	Biofuels Jatropha Program 2007-2012	0	0						
Senegal	2008	SEN08	Lettre de Politique de Développement du Secteur de L’Energie	0	0						
South Africa	2013	ZAF13	The Bio-economy Strategy	11	3	55	13	20	0	39	73
South Korea	2006	KOR06	Biovision 2016 - For Building a Healthy Life and a Prosperous Bioeconomy	0	0						
South Korea	2018	KOR18	Biotechnology in Korea	0	0						
Spain	2016	ESP16	The Spanish Bioeconmy Strategy 2030 Horizon	3	1	100	0	100	0	0	0
Sri Lanka	2010	LKA10	National Biotechnology Policy	0	0						
Sweden	2012	SWE12	Swedish Research and Innovation Strategy for a Bio-based Economy	1	3	100	0	100	0	0	41
Tanzania	2010	TZA10	National Biotechnology Policy	3	2	0	0	100	0	0	0
Tanzania	2014	TZA14	Biomass Energy Strategy (BEST) Tanzania	7	3	53	42	85	0	0	44
Thailand	2019	THA19	Bio-Circular-Green Economy (BCG) in Action: The new Sustainable Growth Engine	6	2	51	73	100	0	0	100
Uganda	2008	UGA08	National Biotechnology and Biosafety Policy	4	1	0	100	100	0	0	100
Uganda	2018	UGA18	Biomass Energy Strategy (BEST)	6	4	20	62	27	8	11	100
United Kingdom	2018	GBR18	Growing the Bioeconomy	3	1	19	0	0	0	81	19
United States	2016	USA16	Federal Activities report on the bioeconomy	0	0						
United States	2016	USA16	Strategic plan for thriving and sustainable bioeconomy	4	1	100	0	0	0	0	0
Uruguay	2012	URY12	Plan Sectorial de Biotechnología 2011-2020	0	0						

Appendix 2: Resilience Statistics Averaged Across Different Characteristics

Appendix 3: Coding Scheme

**Table:**

Resilience to what?
Economic
Basic Goods Security
Water Security
Energy security
Food security
Economic Development/Growth (broadly speaking)
Industrial Sector Development
Biorefineries
Aquatic
Agroprocessing
Regional Development
Small-holder-farmer participation/inclusion / farm diversity
Urban Development
Coastal Development
Productivity
Agricultural Productivity
Plant Health
Animal-Related Concerns
Animal Health
Market Development
Market failures
Asymmetric Information
Monopoly/market power/First Movement Advantages
Non-excludibility/approbriability leading to investmen
Informational externalities.
Coordination failure
Dynamic scale economies and knowledge spillovers
Economic Externalities
Commercialisation
BBP demand (un)certainty
BBP demand opportunity
Export Success/Growth/Leadership/Development
Domestic Market Creation/Development
Domestic Market Access
Competitiveness
Bio-Bio competition
Biomass Production Profitability/Competitiveness
Cost Competition between Bio-based Products
Bio-Non-Bio Competition
Competition between Bio- and Non-Bio Renewable Energies
Competitive Bioeconomy VERSUS Fossil-Fuel-Based Economy (generi
Cost Competition between Bio- and Fossil-fuel-based Products
International Competitive Bioeconomy
Foreign Market Access
Foreign (direct) investment
Biomass Management
Land competition
Biomass Supply (un-)certainty
Supply (In-)Dependence on Other Countries
Resource Scarcity
Logistics/Infrastructure
Business Development
New Businesses
Private Sector Capability
Anchor companies
Business Environment
Financial Risk
Financial Capacity/Budget
Incentives
Business climate (generic statement)
Employment
Cooperation
Cooperation of companies across different value chains
Public-Private Cooperation
Research-Industry Cooperation
Cooperation of companies in the same value chain or util
Fossil Resource Scarcity/Finity/Limit
Social
Behavioural Dynamics
Consumer Behavior
Producer behavior
Social Acceptance of BBPS and methods
Social Acceptance of bio-based products
Ethical concerns
Demographic Dynamics
Population Growth
Migration
Urbanization
Culture
Culture/Cultural Capacity
(In)accessibility
Food (In)accessibility
Public understanding/knowledge of bioeconomy
Human Health
Poisoning
Communicable diseases (Vaccines/Epidemics/pandemics)
HIV/AIDs
Non-communicable diseases
Inequality (other subcategories?)
Urban-Rural
Income-Wealth
Environmental
Environmental Concerns
Soil
Water
Forests (as environmental concern explicitly)
Agricultural land (as environmental concern explicitly)
Marine (as environmental concern explicitly)
Pollution
Pesticides
Plastic/Microplastic pollution
Climate Change
Climate threats
Ocean acidification
Frost
Heat
Salinity
Dry Climate
Desertification
Wildfire
Droughts
Rainfalls/Flooding
Climate Change mitigation
Climate Change adaptation
Environmental Sustainability
Protection
Sustainable resource management
Recycling
Common Pool Resources
Standards
Biodiversity
Species
Genetic
Ecosystem health and services
Ecosystem health
Ecosystem functions & services
Biosafety
Political
Regulation
Recognition of pervers incentives, unproductive paths & lock-in
State Intervention in Economy
National Regulatory (un-)certainty
National Regulatory substance
Governance
Measuring/Monitoring
Corruption
(Topic) Broad/Cross-stakeholder consultation
Inter-Authority-Coordination/Cooperation
International Harmonization
Institutions
Strategic Capacity on a domestic level
Political (In)stability/Wars
State capacity for identifying, measuring, and/or monito
Bureaucracy and administration
International Cooperation
International Political Competition
Level Playing Field in Trade
Geopolitical (Outcome) Equality
Geopolitical Process Equality
Research, Innovation & Technology
Technological (Un)Certainty/Gap
Innovation
Innovation capacity
New products/technologies/production techniques
New Technologies
New Products
New Processes
Skilled Labour
Educational/Training Schemes
Indigenous Knowledge
Digitalisation
Research & Development (multi- & inter-disciplinarity
**Resilience of what?**.
Sector
PRIMARY
A. Agriculture, forestry and fishing
01 - Crop and animal production, hunting and related service ac
011 - Growing of non-perennial crops
Mustard
Sugarcane
012 - Growing of perennial crops
Pongamia
Grasses
014 - Animal production
Genetically modified plant-based foods
02 - Forestry and logging
Jatropha
03 - Fishing and aquaculture
Water-related (BBPS)
Coastal
Marine (BBPS)
Wetlands
Water plants
Algae
S eaweed
SECONDARY
C. Manufacturing
10 - Manufacture of food products
16 - Manufacture of wood, cork, straw (no furniture)
Briquettes
Charcoal
17 - Manufacture of paper and paper products
20 - Manufacture of chemicals and chemical products
21 - Manufacture of basic pharmaceutical products and prepar...
2100: Manufacture of pharmaceuticals, medicinal chemical and bo
D. Electricity, gas, steam and air conditioning supply
351 - Electric power generation, transmission and distribution
E. Water supply; sewerage, waste management and remediation act
38 - Waste collection, treatment and disposal activities; mater
Industrial Waste
Household/Municipal Waste
F. Construction
G. Bioenergy
Biocrude oil
Biogas
Biofuels
Biodiesel
Pellets
Bio-butadiene
QUATERNARY
M - Professional, scientific and technical activities
72 - Scientific research and development
721 - Natural sciences Research and experimental development
Biotechnology (explicit)
Bioeconomy at large
N.A. (of what)
**Resilience for what purpose?**
N.A. (purpose)
TOPIC
Economic
Primary Sector Development
Agricultural Development
Forest Economy
Sustainable Economy
Bioeconomic Transformation/Growth (generic)
Sustainable economy/Clean Growth
Green Economy
Circular Economy
Recycling
Bioenergy
Biogas
Biofuels
Jet/Aviation fuel
Biodiesel
Ethanol
Second generation Biofuels
Heat (generic)
Woody fuel
Briquettes
Charcoal
Electricity
Pellets
Basic Goods Security
Water Security
Energy security
Food security
Economic Development/Growth (broadly speaking)
Industrial Sector Development
Biorefineries
Regional Development
Small-holder-farmer participation/inclusion / farm diversity
Regional Development/Growth
Rural Development
Productivity
Forestry Productivity
Agricultural Productivity
Market Development
Market failures
Non-excludibility/approbriability leading to investmen
Dynamic scale economies and knowledge spillovers
Commercialisation
Competitiveness
International Competitive Bioeconomy
Domestic Market Access
Domestic Market Creation/Development
Export Success/Growth/Leadership/Development
Biomass Management
Biomass Supply (un-)certainty
Supply (In-)Dependence on Other Countries
Logistics/Infrastructure
Business Development
New Businesses
Business Environment
Financial Capacity/Budget
Employment
Social
(In)accessibility
Education (In)accessibility
Food (In)accessibility
Energy (In)accessibility
Medicines/Health System (In)accessibility
Water/Sanitation (In)accessibility
Drugs/Medical Supplies (In)accessibility
Quality of life/Societal well-being
Poverty (Reduction)
Human Health
Food safety
Culture
Environmental
Environmental Sustainability
Sustainable/Low carbon future
Protection
Sustainable resource management
Common Pool Resources
Biodiversity
Genetic
Ecosystem health and services
Ecosystem health
Ecosystem functions & services
Pollination
Climate Change
Climate Change mitigation
Environmental Concerns
Forests (as environmental concern explicitly)
Biosafety
Clean energy
Political
International Political Competition
Governance
Bureaucracy and administration
Research, Innovation & Technology
Bioprospecting
Skilled Labour
Innovation
Research & Development (multi- & inter-disciplinarity
Innovation capacity
New products/technologies/production techniques
New Technologies
New Products
New Processes
**What enhances Resilience?**
N.A. (enhance)
INSTRUMENT
Financial/Treasure
Infrastructure
Establishment of specific research facilities
Establishment of pilot and demonstration plants
Promotion/creation of Biorefinieries
Direct support
Input support schemes
Funding of research/innovation projects
Funding of industry/business
Adaptation Funding for Industry/Business
Innovation Funding for Industry/Business
Funding of Vocational Training Schemes
Public procurement programmes
Tax/Fee reductions for producers
Tax/Fee reductions for consumers
Subsidies for producers
Subsidies for consumers
Credit/Loan Schemes
Payment for Ecosystem Services
Indirect support
Import/Export incentives
Communicative/Informative
Creation of public dialogue platforms
National Bioeconomy Websites (Platforms?)
Media Campaigns/Advertisement
Reports
Public (not vocational) Training/Education Programmes
Market Intelligence Collection/Dissemination
Regulative (Authoritative)
Emissions Trade Markets
Licenses
Private und public standards, certifications and labelling
Regulation (generic)
Production Objectives/Quotas
Pigout Taxes
Statal Organization/Coordination
Abolishment/change of impeding instruments
Monitoring/Measuring
Measuring Entity Creation
Inter-Authority/Ministry Coordination
Intergovernmental Coordination
Regular reviews of policies (learning loops)
Regulative Authority Creation
FDI Promotion Agency Creation
Public Enterprise Creation
Creation of international organizations/regimes/partnerships
Bioeconomy Entity/Body
Public-Private Coordination/Cooperation Facilitation
Cooperation of policy, business, academia & civil society
Facilitation of policy, research and industy netw/coop
Facilitation of research networks/cooperation/partners
Facilitation of science and industry networks/cooperat
Facilitation of science and policy networks/cooperatio
Facilitation of industry and policy networks/cooperati
Facilitation of industry networks/cooperation/partners
Grassroot-stakeholder consultation (Instrument)
Broad/Cross-stakeholder consultation (Instrument)
**Time Dimension**
N.A. (time)
threat to future functions/resilience
threat to the development
threat to current bioeconomy

Appendix 4

## Footnotes

1 Because a text segment could be coded with several codes belonging to different categories and subcategories, the accumulated percentages can exceed one hundred percent.

2 Stable existing barriers differ from potential future shocks in their present effect, and from negative trends in the absence of further exacerbation.

3 That is, the chapters explicitly discussing the bioeconomy and related policy areas, such as, biotechnology, biomass, biofuels and bioenergy.
